# The effect of therapeutic horticulture on non-suicidal self-injury in adolescents with major depressive disorder: protocol for a randomized controlled trial

**DOI:** 10.3389/fpsyt.2026.1790759

**Published:** 2026-06-04

**Authors:** Lei Huang, Tian-Dan Hong, Shi-Cong Liang, Zhao-Jun Huang, Yong-Xin Liang, Ling-Fang Shen, Zhi-Chun Xia

**Affiliations:** 1 The Affiliated Brain Hospital, Guangzhou Medical University, Guangzhou, China; 2 Key Laboratory of Neurogenetics and Channelopathies of Guangdong Province and the Ministry of Education of China,Guangzhou Medical University, Guangzhou, China; 3 School of Nursing, Guangzhou Medical University, Guangzhou, China; 4 The First Affiliated Hospital of Jinan University, Guangzhou, China

**Keywords:** adolescent, major depressive disorder, non-suicidal self-injury, randomized controlled trial, therapeutic horticulture

## Abstract

**Background:**

In recent years, the rising incidence and comorbidity of major depressive disorder (MDD) and non-suicidal self-injury (NSSI) among adolescents have emerged as a critical global public health challenge. Existing interventions often face limitations such as low patient willingness and poor adherence, restricting their effectiveness. This situation creates an urgent need to explore effective and acceptable interventions. Therapeutic Horticulture (TH) is a novel treatment that uses plant cultivation and related gardening activities as a therapeutic medium. It engages patients in planting, nurturing, and harvesting plants to achieve therapeutic effects. While it shows promise for adolescents with MDD, its efficacy specifically in adolescents with comorbid MDD and NSSI remains unclear. Therefore, this trial aims to evaluate the effects of TH on NSSI and psychosocial functioning in adolescents with MDD.

**Methods:**

This is a single-center, block-randomized, two-arm parallel randomized controlled trial (RCT) will be conducted at the Brain Hospital Affiliated with Guangzhou Medical University, Guangdong, China. Sixty-six participants will be allocated to an intervention or control group via block randomization. Inclusion criteria are (1): diagnosis of MDD and NSSI per DSM-5 criteria; (2) aged 12–18 years; (3) provision of informed consent; and (4) adequate comprehension, communication, and writing abilities. Exclusion criteria include comorbid other mental disorders, history of serious physical illness, receipt of systematic horticultural therapy within the past three months, allergies, or suicidal risk behaviors.The control group will receive treatment as usual. The intervention group will receive TH in addition to treatment as usual for two weeks. The primary outcome is the severity of NSSI. Secondary outcomes include depression, alexithymia, self-efficacy, sleep quality, and loneliness. All outcomes will be assessed at baseline (T_0_) and immediately post-intervention (T_1_).

**Discussion:**

This study will investigate the application of TH in adolescents with comorbid depression and NSSI. The findings may offer a novel treatment option for this population and provide evidence for future clinical interventions and research.

**Trial registration:**

https://clinicaltrials.gov/study/NCT07115381. ClinicalTrials.gov identifier NCT07115381. Registered on 11 August 2025. Protocol version: 1.0, dated 31 October 2024.

## 1 Introduction

Adolescent mental health is a critical determinant of lifelong well-being and societal development. Among the various mental health challenges, the co-occurrence of major depressive disorder (MDD) and non-suicidal self-injury (NSSI) presents a particularly complex and urgent public health issue. MDD is a mood disorder characterized by persistent and pervasive low mood, diminished interest or pleasure, and anhedonia. It is often accompanied by symptoms such as appetite loss, sleep disturbances, and difficulty concentrating (1). In severe cases, suicidal ideation and behavior may occur. Of significant concern, a global meta-analysis estimates the prevalence of MDD among adolescents to be approximately 8%, with a rising trend (2).

NSSI refers to the direct and deliberate destruction of one’s own body tissue, through acts such as cutting, burning, scratching, or hitting, that occurs without suicidal intent (3). These behaviors are non-lethal and socially unacceptable. Investigations indicate a global prevalence of approximately 17.7% (4) for NSSI among adolescents, with onset often occurring in early adolescence (5). This behavior is particularly prominent in adolescents with MDD, where its lifetime prevalence can be as high as 52% (6). NSSI poses substantial risks. It increases the risk of suicide among individuals with depression more than seven times (7). Beyond causing physical harm, it can exacerbate psychological distress. According to the 2019 Global Burden of Disease data, NSSI ranks third among causes of disability-adjusted life years in individuals aged 10–24 years, imposing a heavy burden on patients, families, and society (8). The co-occurrence rate of NSSI and MDD is estimated to be between 11% and 15% (9). This complex interaction complicates clinical intervention. Patients with this comorbidity often present with more severe depressive symptoms, poorer treatment response, and higher relapse risk (10). Consequently, effective intervention for NSSI is a critical pathway to improving outcomes in adolescents with MDD.

Current primary interventions for NSSI in adolescents with MDD include pharmacological, physical, and psychological treatments. Pharmacotherapy is widely used but requires consideration of side effects, dependency, and potential increased suicide risk in some adolescents (11). Common physical interventions include repetitive transcranial magnetic stimulation (rTMS) and (Electroconvulsive Therapy, ECT). While preliminary research suggests rTMS may be a promising method, its mechanisms are not fully understood, and robust clinical evidence specifically for NSSI is lacking (12). Some studies have also confirmed that ECT can significantly reduce NSSI in adolescents (13). However, this therapy may be accompanied by a series of adverse effects, such as headache and nausea, and therefore requires cautious use (14). Additionally, some patients and their families are reluctant to accept this treatment, and its efficacy for NSSI remains a subject of debate.

The 2022 UK National Institute for Health and Care Excellence (NICE) guideline on self-harm assessment, management, and relapse prevention emphasizes that interventions for NSSI in adolescents with depression require a comprehensive assessment and treatment of comorbidities and psychosocial factors (15). Evidence-based psychological interventions recommended for NSSI in adolescents with depression include cognitive behavioral therapy (CBT) (16), dialectical behavior therapy (DBT) (17), and acceptance and commitment therapy (ACT) (18) and other interventions. While these therapies show efficacy, they are often constrained by high time and financial costs, specialized requirements, and limited service availability (19). Furthermore, given adolescent developmental characteristics, long-term, single-modality treatments may exacerbate negative emotions, reduce treatment acceptance and adherence, and induce resistance, thereby diminishing therapeutic effects (20). Therefore, exploring effective, integrated, psychologically appealing interventions with minimal side effects is of paramount importance.

Horticultural therapy (HT) is defined by the American Horticultural Therapy Association (AHTA) as therapeutic horticultural activities led by a registered therapist to meet individualized treatment goals (21). Therapeutic horticulture (TH) refers to participation in horticultural activities facilitated by a trained professional, using gardening as a therapeutic means to support program goals (21). The key distinction is that HT requires guidance from a registered therapist, whereas TH can be led by other trained professionals. Both approaches have demonstrated positive effects in mental health rehabilitation (22), and the terms are often used interchangeably in research (23). While HT may yield greater benefits, TH is more feasible for widespread application in settings lacking specialized therapists.

TH is an adjunctive treatment method grounded in horticulture. It integrates multiple disciplines, including rehabilitation medicine, psychology, and sociology. It shows promising application prospects in the field of mental health rehabilitation. Existing evidence indicates that TH can effectively improve depression and anxiety, enhance quality of life and social functioning, and restore hope and confidence in adolescents (24, 25). However, TH interventions in previous studies often lacked a systematic and scientific theoretical framework. With increasing scholarly attention, theoretical research in this field has made progress. For instance, Zhang et al. (26) constructed a healing system framework for TH. This framework uses healing landscapes and activities as its core structure and employs sensory stimulation and structured procedures as its fundamental methods. Its mechanism of action primarily involves a synergistic process of sensory stimulation, emotional responses within psychological mechanisms, and neuroimmune modulation within brain mechanisms, mainly through the five senses. It aims to achieve rehabilitation for patients with mental disorders across four dimensions: positive emotion, functional recovery, skill acquisition, and social interaction. This theory provides a solid foundation for designing targeted TH intervention programs. Compared to traditional medical approaches, TH also offers the advantages of being engaging and having minimal side effects. Despite the considerable therapeutic effects observed for TH in adolescent and depressed populations, research on its application specifically for adolescents with comorbid MDD and NSSI remains extremely scarce.

Based on this rationale, we this designed study as a randomized controlled trial (RCT) to evaluate the effects of TH on NSSI and overall psychosocial functioning in adolescents with MDD. The aim is to explore a gentle and effective intervention method and provide scientific evidence for improving NSSI in adolescents with MDD.

## 2 Methods and analysis

### 2.1 Study design

**Figure 1:**
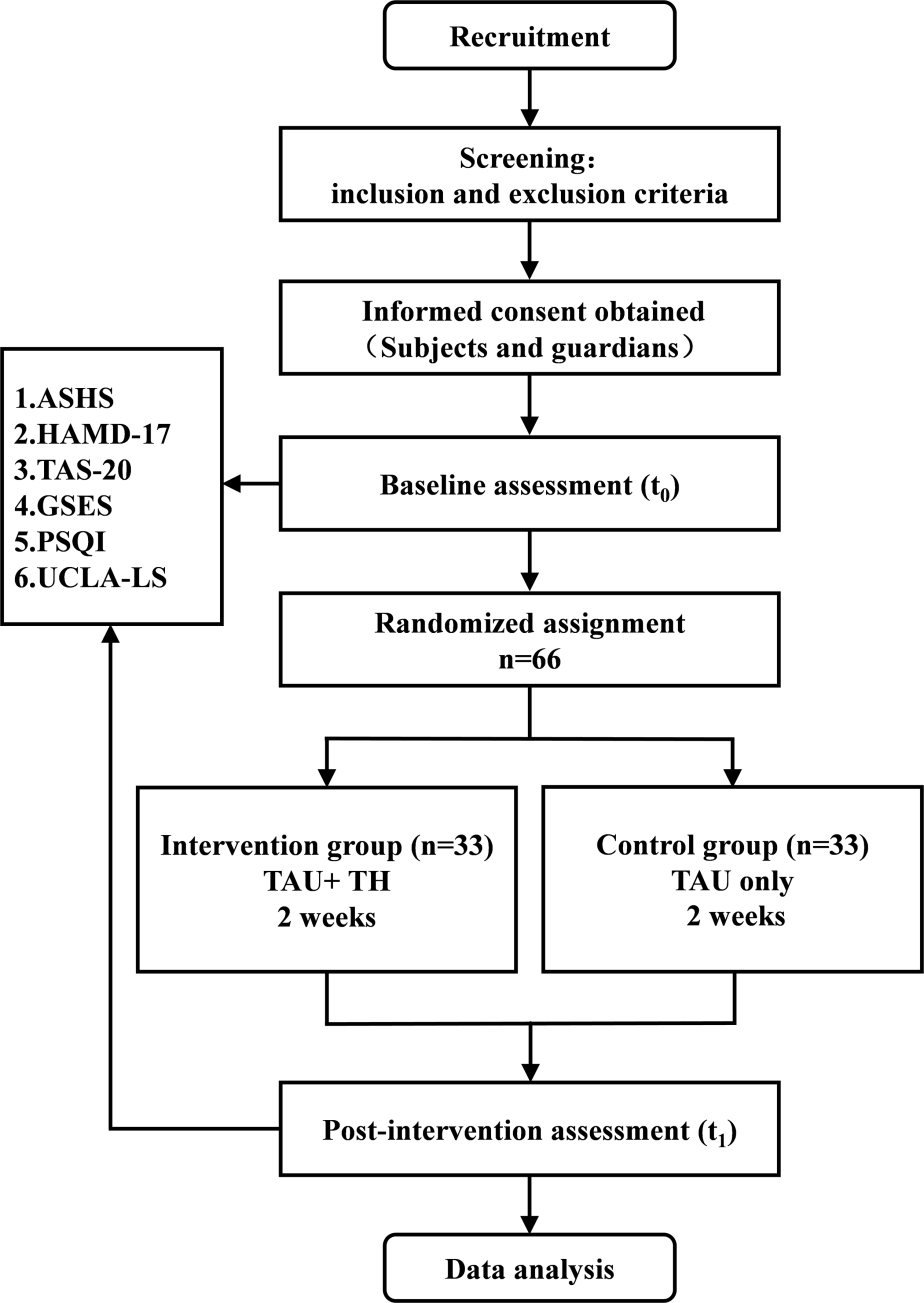
Technology roadmap.

This is a single-center, block-randomized, two-arm parallel randomized controlled trial (RCT). The study protocol adheres to the SPIRIT 2025 statement (27). Participants are allocated in a 1:1 ratio to two groups via block randomization. The control group receives treatment as usual (TAU). The intervention group will receive Therapeutic Hrticulture (TH) in addition to TAU. The intervention period is two weeks. Assessments are conducted at baseline (T_0_) and immediately post-intervention (T_1_). The study flowchart is presented in **Figure 1**, and the schedule of enrollment, interventions, and assessments is detailed in **Table 1**.

**Table 1:** The schedule of enrollment, interventions, and assessments.

Item	Trial period				
	Enrollment		Post-randomization		Closeout
Time point	-T_1_→ T_0_	T_0_	Week 1	Week 2	T_1_
Enrollment					
Eligibility screen	×				
Informed consent	×				
Randomization		×			
Intervention or comparator					
Intervention group (TAU + TH)		×	×	×	×
Control group (TAU only)		×	×	×	×
Assessments					
Demographic information	×	×			
Disease-related information	×	×			
ASHS		×			×
HAMD-17		×			×
TAS-20		×			×
GSES		×			×
PSQI		×			×
UCLA-LS		×			×
Safety evaluation		×			×
Table of trial completion and early termination	×	×	×	×	×

### 2.2 Study setting and participants

#### 2.2.1 Study setting

The study is conducted at the Brain Hospital Affiliated with Guangzhou Medical University, a tertiary Grade A psychiatric specialty hospital. All study procedures, including participant recruitment, intervention delivery, and outcome assessments, take place within the closed psychiatric ward of this hospital.

#### 2.2.2 Inclusion and exclusion criteria

Participants are included in the study if they meet the following criteria (1): Meet the diagnostic criteria for major depressive disorder (MDD) as defined in the Diagnostic and Statistical Manual of Mental Disorders, Fifth Edition (DSM-5); (2) Exhibit characteristics of non-suicidal self-injury (NSSI) as defined in the DSM-5; (3) Are aged between 12 and 18 years; (4) Provide voluntary informed consent from the participant, with additional consent from a legal guardian for those under 18; (5) Possess adequate reading comprehension, verbal communication, and writing abilities.

Participants are excluded from the study if they meet any of the following criteria: (1) Have a comorbid psychiatric disorder such as intellectual disability, autism spectrum disorder, or stereotypic movement disorder with self-injury; (2) Have a history or current diagnosis of a central nervous system disease, endocrine disorder, or other severe physical illness; (3) Are currently receiving or have received any form of systematic horticultural therapy within the past three months; (4) Have a known allergic reaction to pollen, soil, or other materials that may be encountered during the intervention; (5) Exhibit acute suicidal risk or behavior requiring immediate clinical intervention.

#### 2.2.3 Discontinuation criteria

Participants may be withdrawn or discontinued from the study if any of the following criteria are met: (1) Poor adherence or failure to complete study-specified assessments; (2) Incomplete medical records affecting the evaluation of primary or secondary outcomes; (3) Voluntary withdrawal of informed consent by the participant or their legal guardian; (4) Experiencing significant clinical deterioration, serious complications, or special physiological changes during the study that make continued participation unsuitable; (5) The investigator determines, for other justified reasons, that continued participation is not appropriate.

#### 2.2.4 Recruitment

This study plans to recruit 66 adolescent inpatients with MDD and comorbid NSSI at the Affiliated Brain Hospital of Guangzhou Medical University. Trained researchers will conduct an initial screening of potential eligible participants in the system according to the established criteria. Subsequently, the attending physician will review and confirm eligibility based on clinical judgment. Adolescents who meet the inclusion criteria and do not meet any exclusion criteria, along with their guardians, will be invited for a face-to-face interview. During this interview, researchers will provide detailed information and complete the formal informed consent process.

#### 2.2.5 Consent procedure

For participants under 18 years of age, written informed consent is obtained from both the adolescent participant and their legal guardian. The consent procedure is conducted one-on-one by a researcher in a quiet and private setting. The researcher provides detailed explanations about the study’s purpose, procedures, potential benefits and risks, confidentiality measures, the principle of voluntary participation, and the right to withdraw. Ample time is ensured for questions and deliberation. Both the participant and the guardian sign the written informed consent form only after fully understanding the information and without any undue influence. This study does not involve the collection of biological samples. All collected data are used solely for the statistical analyses specified in this protocol.

### 2.3 Randomization and blinding

This study employs block randomization. An independent researcher (the sequence generator), who is not involved in participant recruitment, intervention delivery, or outcome assessment, uses R software (version 4.5.0) to generate the randomization sequence. The block size is concealed and known only to the sequence generator until the trial concludes. Participants are allocated in a 1:1 ratio to either the intervention group or the control group. After generation, the sequence is sealed in sequentially numbered, opaque, sealed envelopes. The custody and opening of these envelopes are the sole responsibility of another independent researcher (the allocation custodian), who is not involved in sequence generation, recruitment, or assessment. Once a participant meets the inclusion criteria and completes both the informed consent process and the baseline assessment, the allocation custodian opens the corresponding envelope in sequential order. The allocation information is then disclosed to the intervention facilitator, and the participant is assigned to the corresponding group to receive the designated intervention.

This study employs a blinded design. Due to the nature of TH intervention, blinding of participants and intervention facilitators is not technically feasible. However, outcome assessors and data analysts remain blinded to group allocation. To minimize the risk of unblinding due to environmental cues in the closed ward setting, the following measures will be implemented. Outcome assessments will be conducted in a designated room that is physically separate from the intervention area and not used for TH activities. Assessment sessions will be scheduled at times that do not overlap with TH sessions. Participants will be instructed not to disclose their group assignment or discuss TH activities during assessments. If accidental unblinding of an assessor occurs, this will be documented, and a different blinded assessor will complete the remaining assessments for that participant. Communication regarding group allocation between researchers and assessors is strictly prohibited. All assessment forms and study documents identify participants only by a study ID, with no group information included. Final unblinding will occur according to the pre-specified study protocol upon trial completion. Additionally, emergency envelopes containing participant allocation and treatment information are prepared in advance. In the event of a serious adverse event (SAE) where the treating physician must know the specific intervention to provide emergency medical care, and following confirmation by the principal investigator, the emergency unblinding procedure will be initiated immediately in accordance with the protocol, with detailed documentation of the event.

### 2.4 Interventions

#### 2.4.1 Treatment as usual

This study aims to evaluate whether adding TH to a standardized regimen of treatment as usual (TAU) provides additional benefit. To independently assess the additive effect of TH as an adjunctive therapy, the control group receives two weeks of TAU during the trial period. All TAU is provided according to the hospital’s standard clinical pathways. TAU comprises treatment and usual care. The treatment consists of antidepressant medication, supplemented by physical therapy, psychotherapy, and rehabilitation training. The usual care includes admission orientation, health education on symptoms, consequences and treatment options for depression and NSSI as well as the importance of treatment adherence, medication guidance, discharge planning, and psychological support.

During the study period, the provision of TAU is at the discretion of the attending physician. Participants are not restricted from receiving other treatments or care beyond the designated protocol. To address potential heterogeneity in TAU, the following TAU components will be systematically recorded for all participants throughout the trial: antidepressant medication, including drug name, daily dose, and any dose adjustments; physical therapy, including type, frequency, and duration; psychotherapy, including modality, number of sessions, and duration; and rehabilitation training, including type and frequency. If a participant’s symptoms worsen and require additional treatment, all related details are fully documented in the case report form. These data will be incorporated into the statistical analysis to control for potential confounding effects.

#### 2.4.2 Therapeutic horticulture

The intervention group will receive the TH intervention based on the healing system framework, in addition to the TAU received by the control group. Specifically, while receiving the identical two-week regimen of TAU as the control group, the intervention group participates in the additional TH program. The TH is delivered in a group format, with 3 to 6 participants per group. Sessions are conducted in the psychotherapy room of the psychiatric ward at the Affiliated Brain Hospital of Guangzhou Medical University. The intervention lasts for two weeks, comprising four sessions per week, each lasting 60 to 90 minutes. The TH protocol was developed by the research team under the guidance of domain experts, based on the healing system theoretical framework. The detailed intervention protocol is presented in **Table 2**.

**Table 2:** Therapeutic Horticulture intervention program.

Session	Activity	Objective
1	1.1 Sensory Immersion & Ice-breaking	-Enhance interest, encourage self-expression, and promote social interaction.
	1.2 Craft Self-made Grass Head Dolls	-Enhance self-efficacy.
2	2.1 Divide & Plant Greenery	-Alleviate stress, enhance self-efficacy, and promote social interaction.
	2.2 Prepare Homemade Orange Peel Seasoning	-Regulate emotions and enhance self-awareness.
3	3.1 Create Simple Pressed Flowers	-Encourage acceptance of outcomes and recognize the growth potential of appropriate pressure.
	3.2 Creative Leaf Art & Printing	-Externalize emotions and reduce emotional repression.
4	4.1 Cooperative Flower Basket Assembly	-Promote social interaction and enhance self-efficacy.
	4.2 Sensory Discrimination of Flowers	-Relieve stress and facilitate emotional expression.
5	5.1 Herbal Tea Tasting	-Relax the body and mind, and focus on the present.
	5.2 Pot Painting & Decoration	-Promote non-verbal self-expression.
6	6.1 Cooperative Nutrient Soil Preparation	-Relieve stress and reduce social avoidance.
	6.2 Combination Potted Plant Arrangement	-Facilitate the expression of inner emotions.
7	7.1 Create Stamping & Embossing Cards	-Enhance self-efficacy, encourage self-expression, and promote social interaction.
	7.2 Craft Self-made Sachets	-Regulate emotions, enhance self-efficacy.
8	8.1 Plant Harvesting & Tea Preparation	-Enhance positive emotions and boost self-efficacy.
	8.2 Achievement Sharing & Reflection	-Strengthen self-efficacy and hope, reduce loneliness, and foster responsibility and connection.

All plant materials used in this program are pre-selected by the research team based on the healing system framework to ensure consistency of sensory stimuli across activities. The plants include aromatic herbs such as mint, patchouli, and lavender; fresh flowers such as spray roses and sunflowers; foliage plants such as fittonia and miniature coconut palm; and edible plants such as citrus fruits and cherry tomatoes. All materials are screened for non-toxicity and potential allergens prior to use. The same core plant materials are used across all intervention groups to ensure standardization. To foster engagement and a sense of ownership, participants may choose from a limited set of options in certain activities, such as selecting preferred flowers for basket assembly or choosing among herbal tea varieties.

To ensure standardization and safety, the TH facilitators must complete standardized TH training through online courses, academic conferences, and practical workshops. They are permitted to deliver the intervention only after mastering the relevant theory, techniques, and practical skills, and upon passing a competency assessment. The principal investigator will assess and supervise adherence to the intervention protocol. All necessary materials are prepared prior to each session. Each session follows a standardized procedure to ensure safety and consistency. Sessions conclude with a summary and sharing segment, during which participants are encouraged to share their experiences and reflections, and the facilitator addresses any questions. Following the session, all materials are inventoried, recorded, and stored properly.

To enhance participant engagement and adherence, the facilitator provides each participant with an activity stamp card. After each TH session is completed, the facilitator seals the activity stamp card. Participants who collect all eight stamps receive a commemorative certificate and a small gift at the final session. Furthermore, the highlights of the next session are previewed verbally at the end of each meeting. The facilitator also provides a reminder for the upcoming session one day in advance.

Adherence to the TH intervention will be quantified as the proportion of scheduled sessions attended by each participant. Completion of at least six of the eight sessions, corresponding to 75% or more, will be considered adequate adherence. Overall adherence will be reported as the mean proportion of sessions attended across all participants in the intervention group, along with the number and percentage of participants meeting the adequate adherence criterion. An exploratory subgroup analysis will be conducted to examine whether the treatment effect on the primary outcome differs between participants with adequate adherence and those with inadequate adherence.

### 2.5 Study outcomes

#### 2.5.1 Primary outcome

The primary outcome is the change in the NSSI level score from baseline to post-intervention. The frequency and resulting harm of NSSI are assessed using the Adolescents Self-Harm Scale (ASHS), a 19-item self-report questionnaire (28). An ASHS total score greater than 0 indicates the presence of NSSI behavior. The NSSI level score is calculated by multiplying its frequency score by its harm score. A higher total score indicates greater NSSI severity (Cronbach’s α=0.93-0.97) (29).

#### 2.5.2 Secondary outcomes

1. Depressive symptom: The 17-item Hamilton Depression Rating Scale (HAMD-17) is the most widely used clinical instrument for assessing depressive symptom severity (30). It consists of 17 items, with a total score ranging from 0 to 52. A higher total score indicates greater severity of depressive symptoms (Cronbach’s α=0.92) (31).
2. Alexithymia: The Toronto Alexithymia Scale (TAS-20) is used to assess alexithymia in individuals (32). It comprises three factors: difficulty identifying feelings, difficulty describing feelings, and externally oriented thinking. The scale consists of 20 items, with a total score ranging from 20 to 100. A higher score indicates greater severity of alexithymia (Cronbach’s α=0.82) (33).
3. General Self-Efficacy: The General Self-Efficacy Scale (GSES) is used to measure general self-efficacy, assessing an individual’s positive self-belief in coping with various life challenges (34). It is a self-report instrument. The Chinese version of the scale has demonstrated good reliability and validity in Chinese adolescent populations. It consists of 10 items, with a total score ranging from 10 to 40. A higher score indicates a higher level of self-efficacy (Cronbach’s α = 0.86) (35).
4. Sleep: The Pittsburgh Sleep Quality Index (PSQI) is used to assess an individual’s recent sleep quality (36). It comprises 19 self-rated items and 5 bed-partner-rated items, of which the 19th self-rated item and all 5 bed-partner-rated items are not scored. The total score ranges from 0 to 21. The first 18 items form seven components: subjective sleep quality, sleep latency, sleep duration, habitual sleep efficiency, sleep disturbances, use of sleeping medication, and daytime dysfunction. A higher total PSQI score indicates poorer sleep quality (Cronbach’s α =0.81) (37).q
5. Loneliness: The UCLA Loneliness Scale (UCLA-LS) is used to assess an individual’s level of loneliness (38). It consists of 20 items, with a total score ranging from 20 to 80. A higher total score indicates a greater sense of loneliness (Cronbach’s α = 0.90) (39).

### 2.6 Data collection and quality control

At the baseline assessment(T_0_), the researcher will collect participants demographic data (age, gender, residence, only-child status, family structure, and family economic status, etc.) and Disease-related information (age of onset, duration of illness, and family history of mental illness, etc.). All outcome measures (primary and secondary outcomes) will be assessed at both baseline (T_0_) and immediately post-intervention (T_1_). These measures comprise the ASHS, HAMD-17, TAS-20, GSES, PSQI, and UCLA-LS.

The following quality control measures will be implemented to ensure standardization and accuracy in data collection and entry:

1. All researchers will receive systematic training to ensure the standardization and consistency of scale administration and data collection.
2. Assessments for each participant at all study time points will be conducted by the same researcher member whenever possible.
3. This trial employs well-established scales and questionnaires with good reliability and validity.
4. Researchers will administer the scales and questionnaires to participants in a quiet, indoor setting using a one-on-one format. Standardized instructions will be provided to explain the scales and questionnaires, ensuring participants correctly and fully understand the items. Upon completion, the scales and questionnaires will be collected immediately. Researchers will then check for completeness. Any missing items will be clarified with the participant and promptly completed.
5. After collection, all scales and questionnaires will undergo initial screening and coding. Data will then be entered into an electronic database using a double-data entry method by two independent personnel. Any discrepancies between the two entries will be adjudicated by a third researcher through verification against the original paper records.

For participants who discontinue the intervention early, every effort will be made to collect all primary and secondary outcome measures at the originally scheduled time point, T_1_. If a participant withdraws consent for further participation but agrees to data use, data collected up to the point of withdrawal will be retained and included in the ITT analysis. Reasons for discontinuation will be documented. In the ITT analysis, missing outcome data from early withdrawals will be handled using multiple imputation as the primary method, with the last observation carried forward used in sensitivity analyses.

### 2.7 Data management and monitoring

All information generated during the trial will be accurately, promptly, and completely recorded in the case report forms (CRF). All data for this study will be de-identified and kept strictly confidential, with access limited to authorized personnel. If a participant withdraws consent for data use, their personal data will be completely deleted. All other data will be stored in anonymized form for at least five years after the publication of results.

Given the short intervention duration, modest sample size, and the low-risk nature of this behavioral intervention, an independent external Data Monitoring Committee (DMC) will not be established. The principal investigator (PI) is responsible for the ongoing monitoring of trial safety and quality, assisted by an independent clinician not involved in trial execution or outcome assessment. When approximately 50% of participants have completed the intervention, the PI will conduct a formal review of safety data, summarizing and analyzing all reported adverse events. The PI retains the ultimate decision-making authority regarding trial continuation. Due to the difficulty of obtaining statistically significant interim results in the early stages, this study will not incorporate any formal interim analyses or pre-specified stopping rules based on efficacy.

### 2.8 Sample size

The primary outcome of this study is the NSSI level score of participants. Let n_1_ and n_2_ respectively represent the sample sizes of the intervention group and the control group. The study sets α = 0.05, β = 0.20 (two-sided test), with a statistical power (1-β) of 80%. The sample size ratio between the two groups is n_1_:n_2_ = 1:1. A previous RCT conducted in Chinese adolescents with comorbid MDD and NSSI, the same target population as this study, was referenced; the primary outcome in that trial was also assessed using the ASHS (40). The mean difference between the intervention and control groups was μ_1_ - μ_2_ = 13, with a standard deviation σ = 17.59. Substituting these values into the formula yielded n_1_ = n_2_ ≈ 29. Accounting for an estimated 10% attrition rate, the final calculated sample size is 33 participants per group, resulting in a total of 66 participants for the study.


$$n=\frac{2\;{\left(Z_{1-\alpha /2}+Z_{1-\beta }\right)}^{2}\;\sigma^{2}}{{\left(\mu_{1}-\mu_{2}\right)}^{2}}$$

### 2.9 Statistical methods

Statistical analysis will be conducted by an independent statistician blinded to group allocation using SPSS 26.0 software. The analysis will adhere to the intention-to-treat (ITT) principle, employ two-sided tests, and consider a P-value of less than 0.05 as statistically significant.

The analysis of outcome measures will utilize three datasets: the full analysis set (FAS), the per-protocol set (PPS), and the safety set (SS). The FAS includes all randomized participants, analyzed according to their originally assigned groups. This is the primary dataset for efficacy analysis. All randomized participants are analyzed in the groups to which they were initially allocated. For missing data in the FAS, the primary outcome will be handled using multiple imputation to generate a complete dataset, upon which the primary analysis will be based. To assess the robustness of the results, a sensitivity analysis will be performed using the last observation carried forward (LOCF) method for imputation. The PPS includes participants with good adherence, who completed the full intervention, and had no major protocol deviations. This set will serve as another sensitivity analysis dataset to verify the stability of the primary analysis results. The SS includes all participants who received at least one dose of the intervention and is used for safety analyses. The evaluation of intervention effects for primary and secondary outcomes will primarily draw conclusions based on the analysis of the multiply-imputed FAS. The results from the LOCF-imputed FAS analysis and the PPS analysis will serve as sensitivity analyses to support the robustness of the conclusions. Safety indicators, such as the incidence of allergic reactions and adverse events, will be analyzed within the SS.

This study will conduct sensitivity analyses to evaluate the robustness of the results. These analyses will include repeating the primary analysis based on the FAS with missing data imputed using the LOCF method, as well as repeating the primary analysis on the PPS. If any baseline variables show imbalance (*P* < 0.1), they will be included as additional covariates in the model, followed by a corresponding sensitivity analysis.

Prior to the intervention, baseline characteristics and baseline outcome measures will be compared between the two groups to assess the balance achieved by randomization. Normally distributed continuous variables will be presented as mean ± standard deviation (SD) and compared using the independent samples t-tests. Non-normally distributed continuous variables will be presented as median and interquartile range (Median, IQR) and compared using the Mann-Whitney U test. Categorical variables will be presented as frequency and percentage (n, %) and analyzed using Chi-square test or Fisher’s exact test.

Analysis of Covariance (ANCOVA) will be used to assess the intervention effects. The post-intervention (T_1_) score will serve as the dependent variable, with the group assignment as a fixed factor and the corresponding baseline (T_0_) score included as a covariate in the model. Analysis of Covariance (ANCOVA) will be used to assess the intervention effects. The post-intervention (T_1_) score will serve as the dependent variable, with the group assignment as a fixed factor and the corresponding baseline (T_0_) score included as a covariate in the model. The primary outcome is a single endpoint and is exempt from multiplicity adjustment. To control for inflation of Type I error due to multiple comparisons across the five secondary outcomes, the Benjamini-Hochberg False Discovery Rate (FDR) correction will be applied to the following outcomes as a family: depressive symptoms measured by HAMD-17, alexithymia measured by TAS-20, self-efficacy measured by GSES, sleep quality measured by PSQI, and loneliness measured by UCLA-LS. A Generalized Linear Mixed Model (GLMM) will be used for a secondary analysis of the primary outcome. A Generalized Linear Mixed Model (GLMM) will be used for a secondary analysis of the primary outcome.

A reduction of ≥30% in the NSSI level score from baseline is prespecified as the minimal clinically important difference (MCID). Participants who achieve this reduction are defined as responders, and the responder rate will be compared between groups using the chi-square test or Fisher’s exact test. For all outcomes, effect sizes (Cohen’s d) with 95% confidence intervals will be reported.

To account for potential confounding arising from heterogeneity in TAU, the following variables derived from the systematically recorded TAU components will be prespecified as covariates and entered simultaneously into the primary ANCOVA model: number of psychotherapy sessions received as a continuous variable, antidepressant dose as a continuous variable, and receipt of physical therapy, defined as rTMS or ECT and coded as a binary variable. Rehabilitation training will be examined descriptively by group; if a between-group difference at *P* < 0.10 is detected, this variable will be added as an additional covariate in sensitivity analyses. When repeating the primary analysis on the LOCF-imputed FAS and the PPS, all prespecified TAU covariates will be retained in the model to verify the robustness of the adjusted treatment effect.

### 2.10 Safety

The pre-defined safety outcomes of this study are the incidence rates of adverse events (AEs) and serious adverse events (SAEs). A serious adverse event (SAE) was defined as any untoward medical occurrence during trial participation that results in death, is life-threatening, requires hospitalization or prolongation of existing hospitalization, or results in persistent or significant disability/incapacity. This includes, but is not limited to, the emergence or clinically significant exacerbation of suicidal or severe self-injurious ideation or behavior during the intervention. A non-serious adverse event is defined as any unfavorable medical occurrence in a participant, regardless of its causal relationship to the intervention.

Researchers will closely monitor participants’ emotional state and safety behaviors throughout the trial. All AEs will be documented in detail on an adverse event form, recording their nature, severity, time of onset, duration, actions taken, and potential relationship to the intervention. In the event of any SAE, the research team will report it to the Principal Investigator and the Ethics Committee within 24 hours. The affected participant will be withdrawn from the study immediately and will receive necessary medical care. The sponsor will provide compensation for any harm directly caused by the trial intervention, in accordance with relevant regulations.

To minimize risks, a corresponding contingency plan will be established prior to the study commencement, and the following measures will be implemented: ensuring a safe environment for activity sessions, guaranteeing the presence of at least one healthcare professional on-site, and equipping the site with a complete first-aid kit and basic emergency equipment. Only safety-designed tools (e.g., rounded shovels, plastic scissors) are permitted. Participants will receive instruction on the proper use and safety precautions of the tools before activities begin. Individuals with a known history of allergy to pollen, soil, or related materials will be excluded during the screening phase. Should any signs of an allergic reaction occur during an activity, the contingency plan will be activated immediately and managed by the on-site healthcare professional.

## 3 Discussion

Current evidence indicates that TH can effectively alleviate stress and mental fatigue in adolescents stemming from academic, interpersonal, or treatment-related pressures (41), demonstrating significant potential for improving adolescent mental health. However, clinical evidence supporting the efficacy of TH specifically for adolescents with MDD and comorbid NSSI remains scarce. Therefore, this study is designed as a RCT to evaluate the effect of TH on reducing NSSI in adolescents with MDD and to observe its impact on psychosocial functioning. The aim is to potentially provide a new pathway for promoting the mental and physical well-being of adolescents.

Therapeutic horticulture is an adjunctive treatment method grounded in the concept of nature-based healing, with horticultural activities serving as its primary medium. According to Attention Restoration Theory (42), natural landscapes and elements allow individuals to experience themselves as part of nature, distance themselves from external pressures, and engage their minds freely without the need for directed attention, thereby facilitating emotional regulation. Nature provides rich multisensory stimulation involving sight, smell, hearing, touch, and taste, which is believed to promote physical and mental health through neural modulation and emotional responses. By guiding individuals to engage with nature, TH helps them gain a sense of self-accomplishment through collaborative and interactive gardening activities. This feature aligns well with the psychological needs of adolescents. Research suggests that some adolescents may feel uncomfortable or find it difficult to express themselves verbally during psychological interventions (43). TH offers a non-verbal, creative platform for communication, potentially reducing the perceived threat for less verbally expressive adolescents.

A strength of this study lies in its use of a non-invasive intervention. Its diverse activity design and engagement strategies are expected to enhance the appeal of the treatment and participant involvement. TH not only supplements existing intervention strategies for NSSI in adolescents with MDD but can also be implemented by trained healthcare professionals. This indicates potential for broad clinical application, particularly in resource-limited settings (19).

This study also has several limitations. First, outcomes were assessed only at baseline and immediately post-intervention. Without follow-up data, whether the effects of TH persist beyond the intervention period remains unknown. Future studies should include at least one follow-up assessment, such as 4 to 12 weeks post-intervention. Second, this single-center trial was conducted in an inpatient psychiatric ward, which, while ensuring standardized intervention delivery, limits generalizability to outpatient and community settings where most adolescents with MDD and NSSI receive care. Third, the sample was restricted to Chinese adolescents, and the findings may not extend to other populations. Fourth, the sample size is powered primarily to detect a moderate-to-large effect; smaller but potentially clinically meaningful effects may not be detected. A future multi-center pragmatic trial incorporating outpatient and community settings, a more diverse sample, and longer-term follow-up is warranted to evaluate the effectiveness of TH in real-world clinical contexts.

## 4 Ethics and dissemination

The sponsor of this study is the the Affiliated Brain Hospital of Guangzhou Medical University (Address: No. 36 Mingxin Road, Liwan District, Guangzhou, Guangdong Province, 510370, P.R. China), which is responsible for the overall trial management, ethical oversight, quality assurance, and participant safety. The study protocol adheres to the principles of the Declaration of Helsinki and has been approved by the Institutional Review Board (IRB) of the Brain Hospital Affiliated with Guangzhou Medical University. Approval Number: (2025) No. (015). Any substantial amendments to the protocol will be documented with reasons, signed by relevant trial parties, and submitted to the ethics committee for approval.

During the design phase of this protocol, informal interviews were conducted with three adolescents with a history of depression and NSSI to gather their perspectives on the format and content of the proposed horticultural therapy activities. Some of their feasible suggestions were incorporated into the final intervention design. However, patients or the public were not systematically involved in the overall design or conduct of the study. As part of our commitment to transparency and respect for participants, a plain-language summary of the main findings will be offered to all participants upon completion of the trial.

The trial results, regardless of outcome (positive or negative), will be disseminated through publication in a peer-reviewed journal. The trial protocol and statistical analysis plan are presented in this article and are also available via the ClinicalTrials.gov registry (ID: NCT07115381). Upon completion of the trial, a summary of the results will be updated on the same registry. Authorship will be determined in accordance with the criteria established by the International Committee of Medical Journal Editors.

## References

[1] Herrman H Kieling C McGorry P Horton R Sargent J Patel V . Reducing the global burden of depression: A Lancet-World Psychiatric Association commission. Lancet. (2019) 393:e42–e3. doi:10.1016/s0140-6736(18)32408-5. PMID: 30482607

[2] Shorey S Ng ED Wong CHJ . Global prevalence of depression and elevated depressive symptoms among adolescents: A systematic review and meta-analysis. Br J Clin Psychol. (2022) 61:287–305. doi:10.1111/bjc.12333. PMID: 34569066

[3] Halicka J Kiejna A . Non-suicidal self-injury (Nssi) and suicidal: Criteria differentiation. Adv Clin Exp Med. (2018) 27:257–61. doi:10.17219/acem/66353. PMID: 29521070

[4] Moloney F Amini J Sinyor M Schaffer A Lanctôt KL Mitchell RHB . Sex differences in the global prevalence of nonsuicidal self-injury in adolescents: A meta-analysis. JAMA Netw Open. (2024) 7:e2415436. doi:10.1001/jamanetworkopen.2024.15436. PMID: 38874927 PMC11179134

[5] De Luca L Pastore M Palladino BE Reime B Warth P Menesini E . The development of non-suicidal self-injury (Nssi) during adolescence: A systematic review and Bayesian meta-analysis. J Affect Disord. (2023) 339:648–59. doi:10.1016/j.jad.2023.07.091. PMID: 37479039

[6] Wu Y Zhang Y Wang C Huang B . A meta-analysis on the lifetime and period prevalence of self-injury among adolescents with depression. Front Public Health. (2024) 12:1434958. doi:10.3389/fpubh.2024.1434958. PMID: 39145175 PMC11322151

[7] Iskric A Ceniti AK Bergmans Y McInerney S Rizvi SJ . Alexithymia and self-harm: A review of nonsuicidal self-injury, suicidal ideation, and suicide attempts. Psychiatry Res. (2020) 288:112920. doi:10.1016/j.psychres.2020.112920. PMID: 32279008

[8] Vos T Lim SS Abbafati C Abbas KM Abbasi M Abbasifard M . Global burden of 369 diseases and injuries in 204 countries and territories, 1990–2019: A systematic analysis for the Global Burden of Disease Study 2019. Lancet. (2020) 396:1204–22. doi:10.1016/S0140-6736(20)30925-9. PMID: 33069326 PMC7567026

[9] Jia Q Wu Z Liu B Feng Y Liang W Liu D . Exploring the longitudinal relationships between non-suicidal self-injury and depressive symptoms in adolescents: A cross-lagged panel network analysis. BMC Psychiatry. (2025) 25:358. doi:10.1186/s12888-025-06806-3. PMID: 40205426 PMC11983942

[10] Yong Y Minlei G Xiaojian Y Huipan W Jinbo S Yuanyuan M . Correlation between adolescent non-suicidal self-harm behaviors and depressive symptoms. Chin J School Health. (2023) 44:659–63. doi:10.16835/j.cnki.1000-9817.2023.05.005

[11] Kennedy SH Lam RW McIntyre RS Tourjman SV Bhat V Blier P . Canadian Network for Mood and Anxiety Treatments (Canmat) 2016 clinical guidelines for the management of adults with major depressive disorder: Section 3. Pharmacological treatments. Can J Psychiatry. (2016) 61:540–60. doi:10.1177/0706743716659417. PMID: 27486148 PMC4994790

[12] Gao J . Repetitive transcranial magnetic stimulation in the treatment of non-suicidal self-injury in depressed adolescents. China J Health Psychol. (2020) 28:1738–43. doi:10.2991/assehr.k.200425.011

[13] Rootes-Murdy K Carlucci M Tibbs M Wachtel LE Sherman MF Zandi PP . Non-suicidal self-injury and electroconvulsive therapy: Outcomes in adolescent and young adult populations. J Affect Disord. (2019) 250:94–8. doi:10.1016/j.jad.2019.02.057. PMID: 30844603

[14] Hu S Xu Y Zhang N . Analysis of risk factors for adverse reactions in patients with depression undergoing modified electroconvulsive therapy. J Med Theory Pract. (2023) 36:1209–11. doi:10.19381/j.issn.1001-7585.2023.07.050

[15] Excellence NIfaC . Self-harm: Assessment, management and preventing recurrence (2022). Available online at: https://www.nice.org.uk/guidance/ng225 (Accessed January 10, 2026).

[16] Liu W Li G Wang C Yu M Zhu M Yang L . Can fluoxetine combined with cognitive behavioral therapy reduce the suicide and non-suicidal self-injury incidence and recurrence rate in depressed adolescents compared with fluoxetine alone? A meta-analysis. Neuropsychiatr Dis Treat. (2022) 18:2543–57. doi:10.2147/ndt.S367931. PMID: 36349346 PMC9637350

[17] Jerome L Ougrin D . Editorial: How can we best support suicidal youth? New evidence for dialectical behavior therapy and different forms of self-harm. J Am Acad Child Adolesc Psychiatry. (2024) 63:860–2. doi:10.1016/j.jaac.2024.05.009. PMID: 38762071

[18] Yuan J Zheng M Liu D Wang L . Effect of acceptance and commitment therapy on emotion regulation in adolescent patients with nonsuicidal self-injury. Alpha Psychiatry. (2024) 25:47–53. doi:10.5152/alphapsychiatry.2024.231324. PMID: 38799501 PMC11114234

[19] Chen R Ma N Wang Y Bai Y Su R Li Z . Current status of psychotherapy and counseling service resources in mental health institutions in Chinese Mainland in 2020: A submitted data-based analysis. Chin J Public Health. (2024) 40:969–73. doi: 10.11847/zgggws1143631

[20] Liu F Zeng Y . Investigation and research on the psychological intervention mechanism for college students. J Fuyang Vocational Tech Coll. (2014) 25:15–8.

[21] American Horticultural Therapy Association . About Ahta definitions and positions (2025). Available online at: https://www.ahta.org/ahta-definitions-and-positions (Accessed January 10, 2026).

[22] Sin PY Li WHC Fan MSN Ng SC Choi KC . Effect of horticultural activities on reducing depressive symptoms in community-dwelling adults: A systematic review and meta-analysis. Int J Nurs Stud. (2025) 168:105081. doi:10.1016/j.ijnurstu.2025.105081. PMID: 40305909

[23] Senting L . Critical concepts for developing theories of horticulture for therapy. Landscape Architecture. (2024) 31:54–74. doi: 10.3724/j.fjyl.202312040544

[24] Oh YA Lee AY An KJ Park SA . Horticultural therapy program for improving emotional well-being of elementary school students: An observational study. Integr Med Res. (2020) 9:37–41. doi:10.1016/j.imr.2020.01.007. PMID: 32071866 PMC7013189

[25] Park KH Kim SY Park SA . Efficacy of a horticultural therapy program designed for emotional stability and career exploration among adolescents in juvenile detention centers. Int J Environ Res Public Health. (2022) 19. doi:10.3390/ijerph19148812. PMID: 35886667 PMC9319874

[26] Zhang S Han Y . Research progress on the application of horticultural therapy in patients with mental disorders. J Chin Urban Forestry. (2021) 19:117–22.

[27] Chan AW Boutron I Hopewell S Moher D Schulz KF Collins GS . Spirit 2025 statement: Updated guideline for protocols of randomized trials. Jama. (2025) 334:435–43. doi:10.1001/jama.2025.4486. PMID: 40294593

[28] Feng Y . The relationship between adolescent self-harm behavior and individual emotional factors and family environment factors. In: . Central China Normal University (2008). Master's thesis.

[29] Shu Z Wang Y Wang X He J . The impact of social exclusion on adolescent self-injury: The longitudinal mediating role of negative emotion. Chin J Clin Psychol. (2025) 33:516–21. doi:10.16128/j.cnki.1005-3611.2025.03.013

[30] Hamilton M . A rating scale for depression. J Neurology Neurosurgery Psychiatry. (1960) 23:56. doi:10.1136/jnnp.23.1.56. PMID: 14399272 PMC495331

[31] Wei L Jie M Wenfeng Q Hongjing S Baiping Y Yunshu Z . The impact of social exclusion on adolescent self-injury: The longitudinal mediating role of negative emotion. Int J Psychiatry. (2025) 52:802–6. doi:10.13479/j.cnki.jip.2025.03.033

[32] Bagby RM Parker JD Taylor GJ . The twenty-item Toronto Alexithymia Scale—I. Item selection and cross-validation of the factor structure. J Psychosomatic Res. (1994) 38:23–32. doi:10.1016/0022-3999(94)90005-1. PMID: 8126686

[33] Xiong Q Shen X Yang H Wang X Yi J Zhu X . Alexithymia and adolescents’ non-suicidal self-injury: The chain mediating of maladaptive emotion regulation and depression. Chin J Clin Psychol. (2023) 3:574–7. doi: 10.16128/j.cnki.1005-3611.2023.03.013

[34] Schwarzer R . Optimistic self-beliefs: Assessment of general perceived self-efficacy in thirteen cultures. World Psychol. (1997) 3:177–90.

[35] Lingkai J Huashan L Xinyu C . Emotional warmth and junior high school students’ psychological resilience: A model of differentiated parenting functions. Educ Res Experimentation. (2023) (6):122–8.

[36] Buysse DJ Reynolds CF Monk TH Berman SR Kupfer DJ . The Pittsburgh Sleep Quality Index: A new instrument for psychiatric practice and research. Psychiatry Res. (1989) 28:193–213. doi:10.1016/0165-1781(89)90047-4. PMID: 2748771

[37] Huipan W Yuan L Xiaojian Y Jinxian W Yi W Yaru G . Association between sleep quality and mental health among middle school students. Chin J School Health. (2025) 46:770–773+8. doi:10.16835/j.cnki.1000-9817.2025173

[38] Pinel EC Long AE Murdoch EQ Helm P . A prisoner of one's own mind: Identifying and understanding existential isolation. Pers Individ Dif. (2017) 105:54–63. doi:10.1016/j.paid.2016.09.024. PMID: 38826717

[39] Yuan R Yang Y Wang Z . The relationship between resilience and non-suicidal self-injury behaviors in adolescents: A moderated mediation model. Chin J Clin Psychol. (2024) 32:843–9. doi:10.1007/s12144-024-06117-y. PMID: 30311153

[40] Ya-Yi Z Xiao-Juan L Ming-Yu L Xue-Ping G Ling-Zhi H . Intervention effect of narrative therapy on non-suicidal self-injury in adolescents with depressive disorder: A prospective randomized controlled study. Chin J Contemp Pediatr. (2024) 26:124–30. doi: 10.7499/j.issn.1008-8830.2308030 PMC10921878 38436308

[41] Guo L Xu W Shi Y Gao S Xiao C Zhang X . Which horticultural activities are more effective for children's recovery from stress and mental fatigue? A quasi-experimental study. Front Psychol. (2024) 15:1352186. doi:10.3389/fpsyg.2024.1352186. PMID: 38680274 PMC11050040

[42] Kaplan S . The restorative benefits of nature: Toward an integrative framework. J Environ Psychol. (1995) 15:169–82. doi:10.1016/0272-4944(95)90001-2

[43] Cameron AY Palm Reed K Gaudiano BA . Addressing treatment motivation in borderline personality disorder: Rationale for incorporating values-based exercises into dialectical behavior therapy. J Contemp Psychother. (2014) 44:109–16. doi:10.1007/s10879-013-9253-9. PMID: 30311153

